# The Colonial Troops Entertainment

**Published:** 1900-12-08

**Authors:** 


### THE COLONIAL TROOPS ENTERTAINMENT.

Lord Abercorn makes the following appeal on behalf of the above object:—

On behalf of the committee appointed to make arrangements for the public reception and entertainment of the representative Colonial Contingent of Forces serving in the war in South Africa, which is expected to arrive in England early next year (as well as of the Canadian Contingent now here), we shall be obliged by your giving publicity, aided by your valuable support, to the appeal which we now make for liberal assistance. A very heavy expenditure must be incurred to give these troops a welcome in some degree worthy of the national appreciation of their splendid services.

The officers and men of these contingents, numbering over 5,000, will be brought to and maintained in this country, and afterwards conveyed to their homes, at considerable expense, the necessary funds being supplied by Parliament, but such funds will not be chargeable with the cost of functions and entertainments not of a strictly military character, nor of the conveyance of the men to provincial cities or other places of interest.

It will be the desire of the people of Great Britain to make the stay of the Colonial Forces in this country as agreeable and instructive as possible, and it is obvious that, as 5,000 men have to be dealt with, this cannot be done without the expenditure of many thousands of pounds.

The committee, therefore, sincerely trusts that a liberal answer will be given to this appeal, in accordance with the universal sentiment of the country, which cordially recognises the patriotic co-operation now volunteered by the Colonies, and the great advance thus made towards the final consolidation of the Empire.

While it is hoped that large subscriptions will be tendered by those who can well afford it, smaller contributions of any amount will be gladly received.

Remittances may be made to the credit of the "Colonial Troops Entertainment Fund," at Messrs. Parr's Bank, Limited, 77, Lombard Street, E.C., or to the Secretary or Treasurer, at 71 & 72 King William Street, E.C.

Her Majesty the Queen, the Prince of Wales, and the other members of the Royal Family, have expressed warm interest in this proposal, and it has also the support of the Prime Minister, the Secretaries of State for the Colonies and for War, the Commander-in-Chief, the High Commissioner for Canada, and the Colonial Agents-General.

A list of the Committee, which is too long to publish, can be seen at the Bankers and at the Office of the Secretary and Treasurer.

I wish to take this opportunity of thanking the Managers of the London theatres and music halls for the prompt and ungrudging manner in which they have responded to the appeal to place at the disposal of my Committee seats for the Colonial troops during their stay in England.

Over 300 free seats at the principal theatres, for every night during the ten days' stay of the Canadian Contingent of about 300 men who arrived on the 28th inst., have with characteristic generosity been given.

